# Clinical characteristics of platelet-mediated killing circulating parasite of major human malaria

**DOI:** 10.1080/07853890.2023.2221453

**Published:** 2023-06-13

**Authors:** Dewu Bi, Xiaodong Huang, Lü Lin, Xike Tang, Yuexi Lu, Zhenxu Lan, Shunda Luo, Jianyan Lin, Xiaocheng Luo

**Affiliations:** a The Fourth People’s Hospital of Nanning, Nanning, China; b Guangxi AIDS Clinical Treatment Center (Nanning), Nanning, China

**Keywords:** *Plasmodium*, malaria, platelets, parasite killing, erythrocytes

## Abstract

**Objective:**

Microscopy was used to characterize platelet-*Plasmodium*-infected erythrocyte interactions in patients infected with *Plasmodium falciparum*, *Plasmodium vivax*, *Plasmodium ovale* or *Plasmodium malariae*, and to investigate the relationship between platelet-associated parasite killing and parasite clearance.

**Methods:**

Data from 244 malaria patients admitted to the Fourth People’s Hospital of Nanning between 1 January 2011 and 30 September 2022, and 45 healthy controls, were collected prospectively and assessed retrospectively. Characteristics of platelet–erythrocyte interactions were visualized by microscopy, and blood cell count and clinical profiles of these participants were obtained from the electronic medical records. ANOVA, contingency tables and Cox proportional hazards regression models were used to do statistical analysis on the subgroups.

**Results:**

Platelet enlargement and minor pseudopodia development were observed. Platelets were found directly attaching to parasitized erythrocytes by all *Plasmodium* species studied, especially mature stages, and lysis of parasitized erythrocytes was connected to platelet-mediated cytolysis. Platelet counts were correlated inversely with parasitaemia and duration of parasite clearance. Artemisinin combination therapy was more effective than artemisinin alone in clearing *Plasmodium* in patients with thrombocytopenia.

**Conclusions:**

Platelet-parasitized erythrocytes cell-to-cell contacts initiated platelet-associated parasite killing and helped to limit *Plasmodium* infection in cases of human malaria. The weakening platelet-associated parasite killing effects could be counteracted by artemisinin combination therapy in patients with thrombocytopenia.

## Summary

Platelets directly attaching to parasitized erythrocytes.

Platelets correlated inversely with parasitaemia and duration of parasite clearance.

Artemisinin combination therapy was more effective than artemisinin alone.

Weakening killing effects may counteract by artemisinin combination therapy.

## 1. Introduction

Human malaria fatality rate has been decreasing at a gradual rate over the last two decades, but it has surged again, exceedingly more over a million in 2020 [1]. Although prior studies suggested a potential pathogenesis in malaria is essentially regulated by host innate and adaptive immune responses [2–4] and parasite biomass [5–7], unfortunately, the clinical characteristics and physiological mechanisms involved in *Plasmodium* pathogenesis are still rarely investigated. Clinically, thrombocytopenia is a common observed symptom in patients infected with malaria [8–10]. Furthermore, an increasing number of studies have reported that parasite density, severity of *Plasmodium* infection and clinical consequences are related to the extent of thrombocytopenia [11]. Collectively, these findings support the hypothesis that platelets may play an important role in the pathogenesis of platelet-associated parasite killing in *Plasmodium*-infected patients.

Platelets, the second most numerous haemocytes of the peripheral circulation, are considered principle to be regulators in haemostasis and thrombosis. However, there is an increasing awareness that platelets are critical contributors to innate and adaptive immunity [12,13], since platelets could coordinate host immune responses by secreting immunoregulatory molecules and interacting with other cells [9,14], and they may play a host-protective function in infectious diseases [14]. Platelet granules deposit numbers of defence peptides and immunomodulatory molecules and released after platelet activation. Platelet-derived antimicrobial molecules have the broad-spectrum activities against pathogens [9,15–19], and platelet factor 4 (PF4) is the first platelet-derived host defence peptide discovered as killing the malaria parasite [12]. Hence, platelets are key immunological contributors in infectious diseases caused by the main *Plasmodium* species in the vasculature.

Platelets bind to parasitized haemocytes and kill the parasite therein, inhibiting the proliferation of intraerythrocytic *Plasmodium*. Both the Duffy-antigen (Fy) [20,21], a chemokine receptor synthesized by erythrocytes, and PF4 [20,22], an extensive antimicrobial peptide released by platelets, are required for the eradication of the major *Plasmodium* species by platelets. A mechanism of platelet-mediated direct killing of parasites incorporates the secretion of PF4 by platelets in proximity to parasitized erythrocytes, which then enters the *Plasmodium*-infected red blood cells (iRBCs) through the Fy and kills intraerythrocytic parasites [9,20,23]. Platelet–iRBC direct cell-to-cell contact is an initiating factor and a significant driver of platelet-associated parasite elimination.

There are currently no clinical investigations evaluating clinical characteristics in platelet–iRBC cell-to-cell contacts in patients infected with *P. ovale*, and *P. falciparum*, *P. vivax* or *P. malariae* are not incompletely characterized. In this study, we characterized the platelet–iRBC cell-to-cell interaction in individuals infected with *P. falciparum*, *P. vivax*, *P. ovale* or *P. malariae*, and investigated the association between platelet-mediated parasite killing and parasite clearance.

## 2. Materials and methods

### 2.1. Study design and participants

This study was a retrospective examination of prospectively collected data from patients, who were admitted to the Fourth People’s Hospital of Nanning between 1 January 2011 and 31 August 2022, and whose malaria infection had been verified. The current study only included patients who were infected with malaria. The following were the inclusion criteria. (i) Patients who have been received a malaria infection diagnosis. (ii) Within 48 h of their symptoms showing, patients were admitted to the hospital. (iii) Patients were admitted to the hospital for chemotherapy and up to blood smear negativity for three successive measures, each separated by 24 h, or the elimination of all clinical evidence of malaria for at least three days with no ongoing symptoms and signs of the disease.

This was a retrospective case series study, and no patients were actively engaged in the study design, research questions or outcome measures. No patients were requested to provide interpretation or write up of results. This study was conducted according to the guidelines of the Declaration of Helsinki and approved by The Ethics Committee of The Fourth People’s Hospital of Nanning (No. [2019]39, [2020]24, [2021]23).

### 2.2. Diagnostic categories

Venous blood samples for microscopic and routine blood tests were taken at the same time, with 24–48 h between each collection for the microscope and routine blood count. The initial identification of malaria species and parasite density were based on microscopic analysis of Giemsa-stained thin and thick blood smears, then nested PCR testing verified malaria parasites and identified species.

### 2.3. Laboratory procedure

Venous blood samples (4 mL) were collected using EDTA-anticoagulant tubes from all cases. Thick and thin blood smears were prepared using the manual wedge technique and were stained with Giemsa stain using Giemsa stain-blood cell/Plasmodium staining kit (BaSO, Zhuhai, China) according to the manufacturer’s instructions. Circulating parasitaemia was determined with thick and thin blood films under light microscope with ×1000 magnifications and imaging was performed using the digital microimaging system with BX43F software (MshOt, Guangzhou, China). Routine complete blood cell counts were performed using the automatic blood cell analyser XN-9000 (Sysmex, Kobe, Japan).

### 2.4. Sample size calculation

A power analysis was conducted based upon pre-experiments. The main findings showed that a cohort of 142 individuals with confirmed *P. falciparum*, 61 with *P. vivax*, four with *P. malariae* and 37 with *P. ovale* infection provided the mean of platelets and standard deviation (SD) (106.4 ± 72.66) (×10^9^/L), (101.0 ± 58.69) (×10^9^/L), (82.5 ± 28.34) (×10^9^/L) and (107.9 ± 54.25) (×10^9^/L), respectively. Null hypothesis value (74.2 × 10^9^/L) was available in the literature [24–26]. To attain a 95% confidence interval with a two-sided type I error of 5% and a statistical power of 80%, 42 participants for *P. falciparum*, 40 for *P. vivax*, 94 for *P. malariae* and 23 for *P. ovale* were required, respectively.

### 2.5. Statistical analysis

Normally distributed continuous variables are presented as mean and SD, and non-normally distributed variables as median and interquartile range (IQR). Categorical variables are presented as count (%). The means of continuous variables were compared using independent sample *t*-tests when the data were normally distributed; otherwise, used the Mann–Whitney *U*-test. Survival analysis was performed using the Kaplan–Meier survival and Cox model analysis. Statistical analyses were conducted using GraphPad Prism software (version 8.0) (La Jolla, CA) and MedCalc^®^ statistical software (version 15.8) (Ostend, Belgium). For all statistical tests, *p* < .05 was considered to indicate statistical significance.

## 3. Results

### 3.1. Baseline clinical features

Between 1 January 2011 and 31 October 2022, 293 participants, 244 patients with malaria and 49 healthy controls, were included in this study; the median age was 37 years (IQR 31–45) and 259 (88%) participants were male (Table 1). The most frequent clinical symptoms and comorbidities are summarized in Table 1. Of 244 cohort patients, 199 (82%) suffered from febrile, 174 (71%) exhibited shivering and 113 (46%) presented fatigue or asthenia. Fifteen (6%) cohort patients with viral hepatitis were reported and 12 (5%) co-infected *Fasciola hepatica*.

**Table 1.** Baseline characteristics of patients with malaria and healthy control patients.

	Controls, *n* = 45	*P. falciparum*, *n* = 142	*P. vivax*, *n* = 61	*P. malariae*, *n* = 4	*P. ovale*, *n* = 37	*p* Value
Demographics						
Age, years	35 (30, 45)	40 (32, 46)	35 (30, 44)	30 (30, 35)	39 (32, 42)	.3121
Male, *n* (%)	40 (89%)	129 (91%)	49 (80%)	4 (100%)	37 (100%)	–
Routine blood						
HGB (g/L)	149 (136, 157)	132 (114, 150)	139 (124, 151)	131 (119, 153)	138 (128, 150)	.0003
RBC (×10^12^/L)	5.09 (4.62, 5.42)	4.68 (4.09, 5.11)	4.83 (4.40, 5.27)	4.53 (4.05, 5.17)	4.87 (4.31, 5.14)	.0006
WBC (×10^9^/L)	7.34 (5.61, 8.82)	6.64 (4.94, 8.61)	6.86 (5.51, 8.46)	6.53 (5.59, 8.19)	6.80 (6.35, 8.28)	.7201
LYMPH (×10^9^/L)	2.29 (1.99, 2.80)	1.02 (0.58, 1.47)	1.14 (0.58, 1.69)	0.98 (0.44, 1.49)	1.25 (0.92, 1.59)	<.0001
PLT (×10^9^/L)	223.5 (185.3, 260.5)	92.5 (52.8, 136.3)	91.5 (57.0, 134.8)	84.5 (55.0, 108.0)	100.0 (69.5, 138.5)	<.0001
PDW (fL)	16.0 (15.7, 16.3)	15.9 (14.0, 16.4)	16.0 (15.4, 16.5)	16.4 (12.8, 17.7)	16.1 (15.6, 16.5)	.1564
MPV (fL)	8.7 (8.2, 9.5)	10.1 (9.3, 11.4)	10.0 (8.5, 10.8)	10.3 (9.1, 11.7)	9.4 (8.8, 10.5)	<.0001
Comorbidities						
Diabetes	–	4 (3%)	4 (7%)	0 (0%)	0 (0%)	–
Hypertension	–	3 (2%)	0 (0%)	0 (0%)	1 (3%)	–
Virus hepatitis	–	11 (8%)	2 (3%)	0 (0%)	2 (5%)	–
COVID-19	–	0 (0%)	0 (0%)	0 (0%)	1 (3%)	–
*Fasciola hepatica*	–	6 (4%)	3 (5%)	1 (25%)	2 (5%)	–
G6PD deficiency	–	6 (4%)	4 (6%)	0 (0%)	3 (8%)	–
Clinical signs						–
Fever	–	117 (82%)	53 (87%)	4 (100%)	25 (68%)	–
Shivering	–	99 (70%)	47 (77%)	4 (100%)	24 (65%)	–
Headache	–	63 (44%)	27 (44%)	2 (50%)	13 (35%)	–
Muscle soreness	–	12 (8%)	7 (11%)	1 (25%)	7 (19%)	–
Fatigue/asthenia	–	65 (46%)	36 (59%)	1 (25%)	11 (30%)	–
Nausea/vomiting	–	23 (16%)	6 (10%)	0 (0%)	4 (11%)	–
Cough	–	18 (13%)	9 (15%)	4 (100%)	3 (8%)	–

Data are in median (IQR) or *n* (%). Some patients had multiple comorbidities and clinical signs. One-way analysis of variance (ANOVA) was conducted by Brown–Forsythe and Welch ANOVA tests. Lymphopaenia and thrombocytopenia are defined as a lymphocyte count <1.1 × 10^9^/L and platelet count <100 × 10^9^/L, respectively.

Compared with healthy controls, haemoglobin (HGB) and red blood cell (RBC) were significantly lower in patients with malaria at enrolment. In total, 45 (31%) cases with falciparum malaria presented anaemia, 11 (18%) was reported in *P. vivax* patients and five (13%) in *P. ovale*. As compared to the non-falciparum malaria infections, patients with *P. falciparum* infection were more significantly susceptible to anaemia, the relative susceptibility was 1.90 (95%CI 1.06–3.54, *p* = .021). In addition, common grades in patients with malaria in this cohort included lymphopaenia and thrombocytopenia, and cumulative incidence of lymphopaenia and thrombocytopenia were 116 (48%) and 125 (51%), respectively. Thrombocytopenia was reported in more than half of cases; however, routine blood analysis coincided with a significant enlargement of the mean platelet volume (MPV).

### 3.2. Platelet–iRBC complexes occur in peripheral blood of patients with malaria

**Figure 1.**
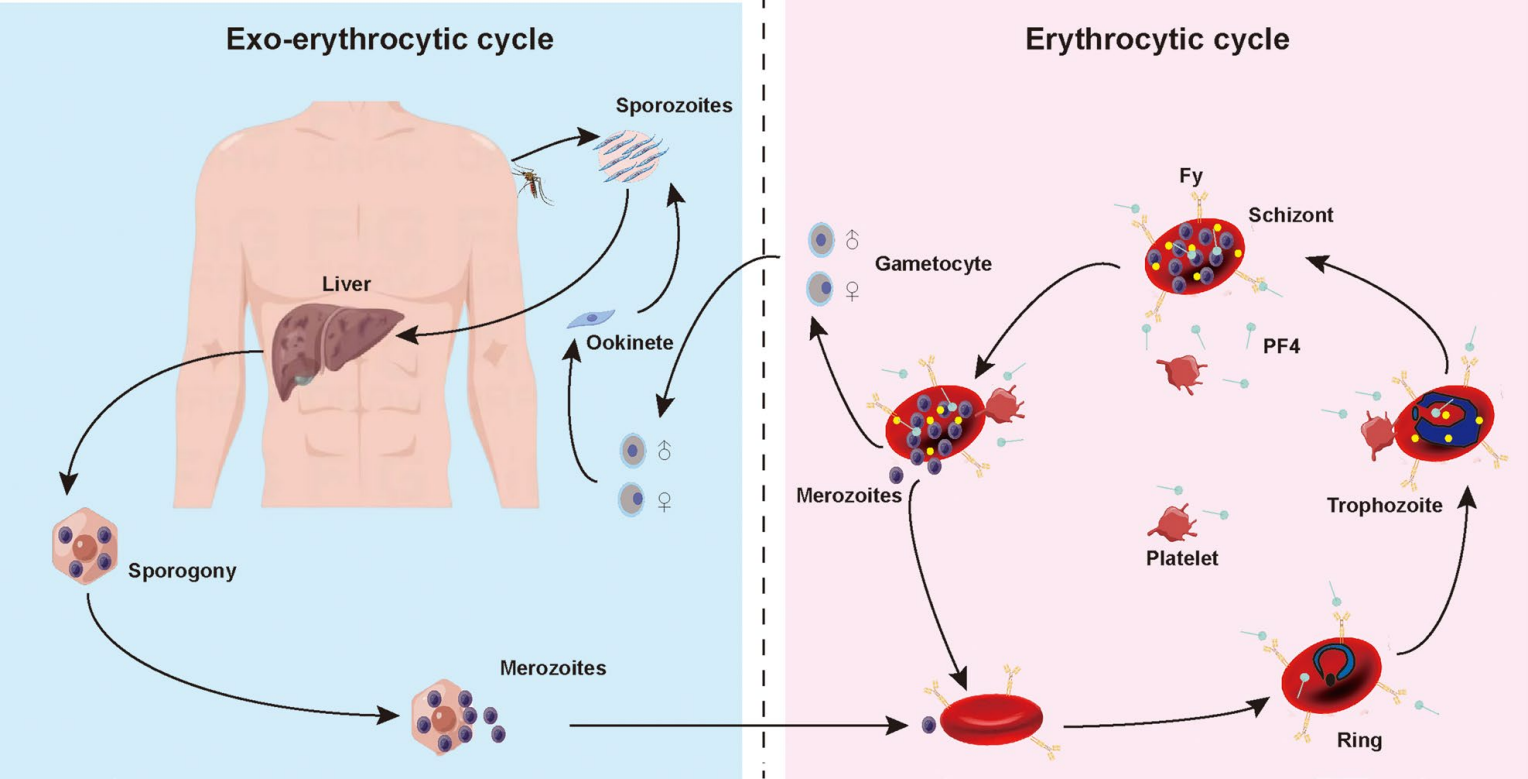
The life cycle of *Plasmodium spp* and platelet-associated parasite killing during erythrocytic stage. Platelet factor 4 (PF4) was released by platelets following cell-to-cell contacts with *Plasmodium*-infected red blood cells (iRBC). PF4 enters the cell via Duffy-antigen (Fy) and kills *Plasmodium* directly.

A mechanism of platelets kills circulating malaria parasites via binding to *Plasmodium*-iRBCs and directly killing the cycling parasites of all important *Plasmodium* species (Figure 1). Platelet-mediated parasite killing encompasses PF4, a CXC-type chemokine secreted by activated platelets from intracellular a granules, that, on crossing through membrane to permeate into erythrocytes via Duffy antigen (Fy), a receptor for chemokine and malaria parasites expressed by erythrocyte, kills *Plasmodium* species by inducing selective cytolysis of the parasite digestive vacuole. Sequestration of platelet-associated to parasitized erythrocyte is a critical mechanism in enabling platelet activation, which subsequently results in the release of an abundant component of PF4. The *Plasmodium* lethal impact of PF4 is exerted dependent upon Fy molecular, which binds PF4.

**Figure 2.**
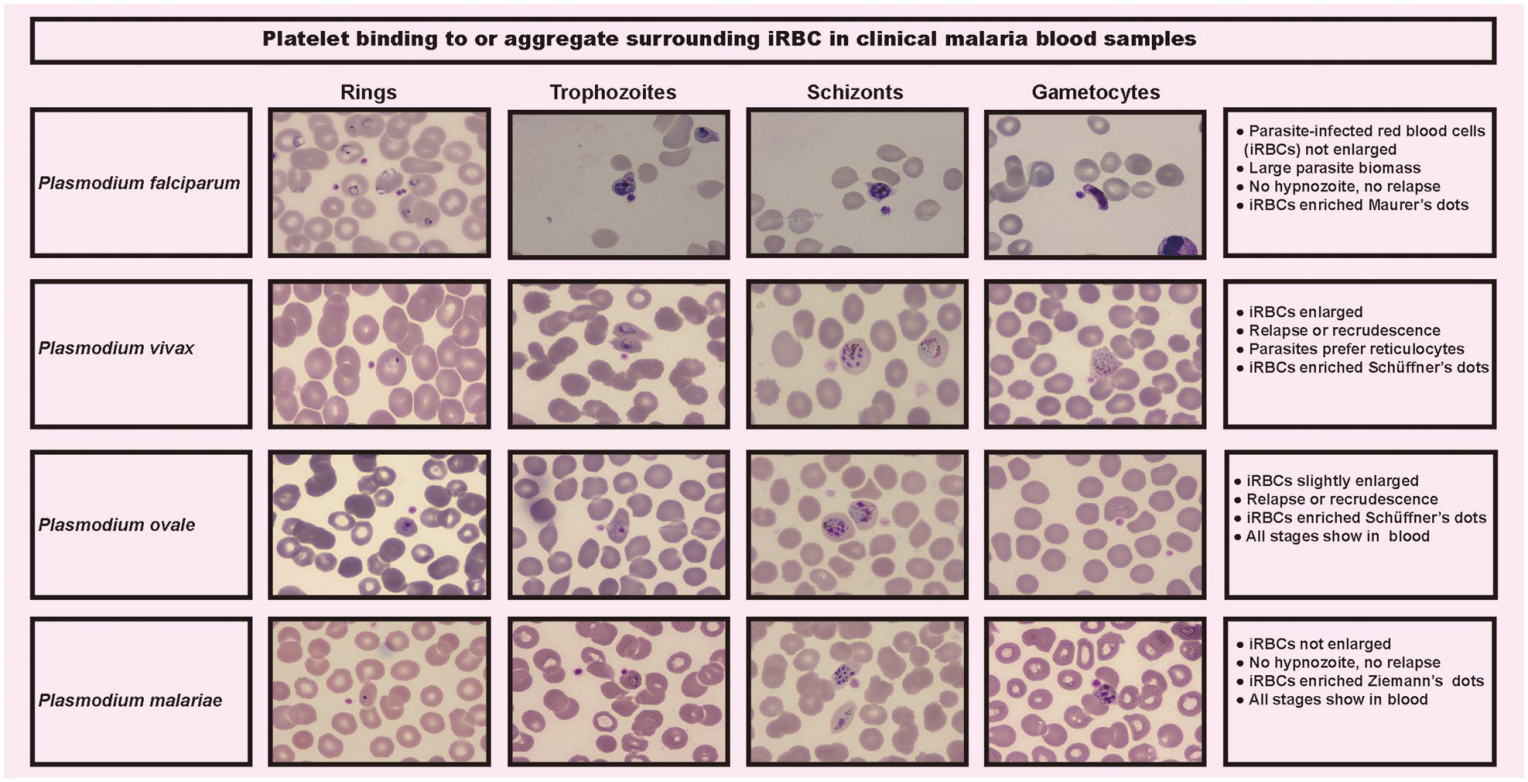
Platelets attach to iRBC in clinical malaria peripheral blood specimens. Platelet-bound iRBC images were captured from a thick blood smear stained with Giemsa at ×1000 magnification utilizing the digital microimaging system with MshOt software.

**Figure 3.**
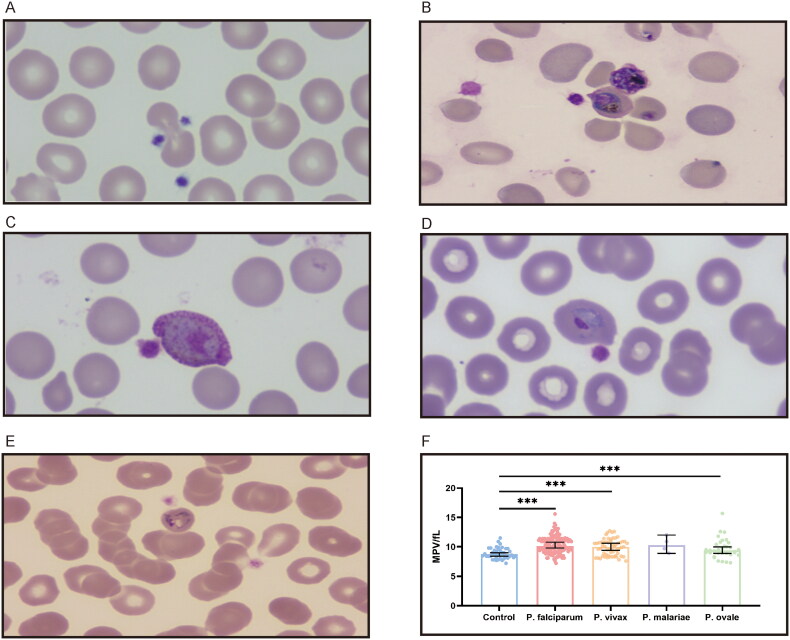
Platelets enlarge in apparent size in the formation of platelet–iRBC aggregates. (A) Platelets adhere to uninfected erythrocytes in healthy controls. (B) Platelets bind to *P. falciparum*-infected erythrocytes at the ring stage. (C) Platelets attach to erythrocytes infected with *P. vivax* at the gametocyte stage. (D) Platelets cling to erythrocytes infected with *P. ovale* at the trophozoite stage. (E) Platelets bond to *P. malariae*-infected erythrocytes at the trophozoite stage. (F) Platelets volume comparison.

**Figure 4.**
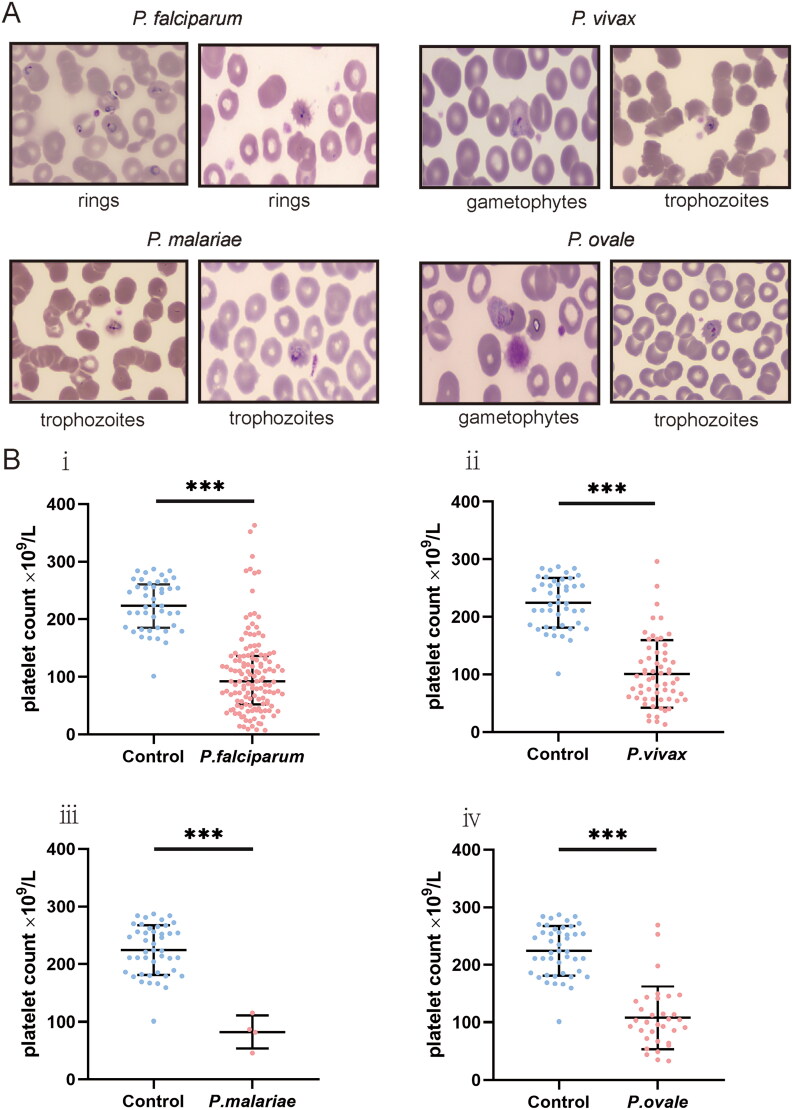
Platelet-associated parasite killing in all four major Plasmodium species. (A) Cytotoxic effect mediated by platelets. (B) Platelet counts comparison.

A microscopic examination of 244 patients was performed. Platelets binding to iRBC and uninfected red blood cells (uRBC) were seen in Giemsa-stained thin blood smears, with platelets appearing to prefer adhering to iRBC or clearly formed aggregates surrounding the iRBC (Figure 2). Platelets were found to be attached to or evident surrounding all asexual parasite stages in major *Plasmodium* species. *P. falciparum*–iRBC complexes were more facilitated to be observed during the ring phase of the *P. falciparum* lifecycle. *P. vivax*, *P. ovale* or *P. malariae*–iRBC complexes, in contrast, were easier to detect at the more mature stages. Platelet size increased in platelet–iRBC complexes compared to controls (Figure 3). Such a finding is consistent with the routine blood analysis. Platelet cytoplasm was stained pink in patients, while it was tinted mauve in healthy controls. Giemsa-staining showed that the margins of the platelets in patients were somewhat fuzzy and not strongly demarcated, with some long filopodia-like protrusions protruding from the edges on a frequent basis. Evidence of platelet-associated cytotoxicity against iRBC was presented (Figure 4(A)). Of malaria patients, there was significant lysing in iRBC, which contact with platelets was connected to cytotoxic activities. In contrast, RBCs from platelet–RBC complexes did not lyse in healthy control cells.

### 3.3. Correlation between platelets and parasite clearance

In malaria patients, thrombocytopenia was one of the most frequent treatment-related adverse events (Figure 4(B)). A statistically significant inverse correlation between platelet levels and parasitaemia (*r* = –0.355, [95%CI −0.462, −0.237], *p* < .0001) was observed in patients infected with malaria. As compared to patients, of whom had *P. falciparum* infection, with normal-levels of platelet, patients with lower-levels of platelet had a longer duration of parasite clearance (median duration: 7 [95%CI 6–8] vs. 9 [95%CI 7–9] days, *p* = .0343), and platelet levels were associated with risk of longer length of parasite clearance (HR = 0.7031, [95%CI 0.475–1.041]). Patients, of whom had *P. vivax* infection, with a low-levels of platelet, needed a longer duration for parasite clearance than those with a normal-levels of platelet (median duration 7 [95%CI 5–8] vs. 5 [95%CI 4–7] days, *p* = .0206), and platelet levels were associated with risk for longer parasite clearance periods (HR = 0.5853, [95%CI 0.3226–1.062]). Cox proportional hazard regression analysis was not performed in patients with *P. ovale* and *P. malariae* infection for risk and duration of parasite clearance because of the small sample size.

There were 131 patients treated with the artemisinin-derivatives artesunate, artemether or dihydroartemisinin alone. These patients who suffered from thrombocytopenia had a longer duration for parasite clearance than those with a normal-levels of platelet (median duration 8 [95%CI 3–12] vs. 6 [95%CI 4–8] days, *p* = .0363), and platelet levels were associated with risk of longer length of parasite clearance (HR = 1.621, [95%CI 1.031–2.549]). Another 61 patients were treated with the artemisinin-derivatives artesunate, artemether or dihydroartemisinin plus amodiaquine, primaquine or chloroquine; however, patients with a low-levels of platelet seem to prefer shorter duration for parasite clearance than those with a normal levels of platelet (median duration 6 [95%CI 4–7] vs. 7 [95%CI 5–8] days, *p* = .0369), and platelet levels were associated with risk of shorter length of parasite clearance (HR = 0.5100, [95%CI 0.2709–0.9600]).

There were 110 patients with thrombocytopenia, and 78 patients were treated with the artemisinin-derivatives artesunate, artemether or dihydroartemisinin alone, and the remaining patients were treated with artemisinin combination therapy. Patients, inflicted with thrombocytopenia, were treated with the artemisinin-derivatives alone had a longer duration for parasite clearance than those with artemisinin combination therapy (median duration 7 [95%CI 5–8] vs. 6 [95%CI 4–7] days, *p* = .0355), and the artemisinin combination therapy was associated with the risk of shorter length of parasite clearance (HR = 0.5605, [95%CI 0.3269–0.9615]). However, the difference of duration for parasite clearance between the artemisinin-derivatives alone and the artemisinin combination therapy group (median duration 7 [95%CI 5–8] vs. 7 [95%CI 5–8] days, *p* = .1454) in patients with normal-levels of platelet was not observed.

## 4. Discussion

Here, we show for the first time in humans the clinical characteristics by which platelet-associated PF4 aids in the suppression of intra-erythrocytic malaria parasites, a circulating microbial pathogen. Platelets were found attached to *Plasmodium*-iRBCs from all the four main *Plasmodium* species: *P. falciparum*, *P. vivax*, *P. oval*e or *P. malariae*. Platelets were also observed bound to uRBCs, while platelet-uRBC complexes were significantly lower than the proportions of platelet–iRBC. Furthermore, the number of platelets was shown to be negatively elated to parasite biomass, implying a possible cause and effect relationship between platelet-associated parasite killing and parasite load. Besides, evidence of platelet cytotoxic cell killing action against iRBC was detected utilizing a microscope analysis of blood smears. The prevalence of platelet–iRBC complexes in the vicinity of malaria cases, the cytotoxicity of platelets against iRBC, and the inverse relationship between platelets and parasitaemia point to the significance of platelets in the host’s capability to control parasites.

Previous studies suggest a possible mechanism of PF4 aggregation and parasite demise via direct platelet–iRBC interaction, followed by proximal PF4 secretion and entry into the parasite using the Duffy-antigen [20–23]. Although number of platelets present at the platelet–iRBC complexes, a substantial proportion of platelets are much more broadly distributed in the area surrounding iRBCs, we speculate that platelet cytoadherence to iRBC is not robust and platelet–iRBC complexes are separated by mechanical force during blood smear preparation. In addition, platelet volume enlargement and the co-occurrence of filopodia-like structures were also observed in patients, these changes indicate that platelet alterations may contribute to the formation of platelet–iRBC complexes and help to PF4 secretion from platelet and achieve higher efficiencies in entry of PF4 into iRBCs.

Platelet cytoadherence to endothelial cells and white blood cells are well-known pathophysiological causes of vascular and inflammatory disorders [14], while the incidence of platelets contacting erythrocytes in any illness condition has been unidentified. We systematically described the clinical characteristics of platelet-associated parasite killing and focused upon the features of the interaction between platelets and iRBC. Our combined results showed that platelets exhibit a better capacity to bind *Plasmodium*-iRBC. Collectively, platelets preferentially bind to mature asexual stage parasites, consistent with previous study. Affinity between platelets and *Plasmodium*-iRBC might be partly influenced by the proteins, expressed by parasite, on the erythrocytes surface. For example, *P. falciparum* adhesion molecule PfEMP1 mediated platelet interaction through CD36 [27], while *P. vivax* reticulocyte-binding protein 2b (P*v*RBP2b) regulated platelet contact via transferrin receptor 1 (TfR1) [28], and platelet attachment to uRBC may be coordinated by erythropoiesis ICAM-4 and platelet GPIIb/IIIa [29]. It is yet unknown what additional RBC and platelet molecules, particularly those from different *Plasmodium* species, are and what functions they play in platelet–RBC interaction.

Widespread platelet activation, immune-mediated exhausting and vascular pooling have all been proposed as reasons of thrombocytopenia [9]. Platelet–RBC complexes may be linked to malaria-induced thrombocytopenia, as previously described [9], and confirmed in our investigation. Platelets are not identified by haematological analysers when platelet–RBC complexes develop, thus the development of platelet–RBC complexes would result in visible platelet decrease. Patients with a larger parasite biomass have lower platelet counts, which is congruent with clinical data. Platelet counts were shown to be negatively related to parasite clearance time, indicating a possible impact link between platelet-associated parasite killing and parasite growth.

Platelet counts were associated with the implementation of highly efficient antimalarial therapeutic interventions. Platelet depletion significantly attenuated platelet-associated parasite killing, weakening effects became particularly evident in the patients with thrombocytopenia were treated with artemisinin-derivatives artesunate, artemether or dihydroartemisinin alone. However, the weakening impacts could probably be counteracted by the artemisinin-derivatives artesunate, artemether or dihydroartemisinin plus amodiaquine, primaquine or chloroquine in patients with thrombocytopenia.

Therapies with high doses of artemisinin derivatives somehow does not reduce deaths or neurological disability in the majority of cerebral malaria cases [30]. Given the parasite resistance, quinoline derivatives are even less efficient at treating severe malaria than artesunate and artemisinin derivatives [31]. However, quinoline derivatives are common rapidly schizonticidal substances, which are able to reduce the mortality and mortality and alleviate the neurological cognitive deficits in cerebral malaria patients [30]. As a result, the World Health Organization has recommended combining quinoline derivatives with artemisinin-based combination therapies (ACTs). Artemisinin and ACT partner-drug resistant parasites have spread from southeast Asia to sub-Saharan Africa and Latin America, posing serious challenges to malaria control [32–34]. The diffusion of artemisinin and ACT partner-drug resistant parasites drives an urgent investigation for novel therapies, especially cerebral malaria. Computer-aided drug design (CADD) and high-throughput screening (HTS) are the two most promising methods for developing novel treatments and could meet the urgent demand for treatment of malaria and should be used in the pharmaceutical industry [35–36].

Duffy-antigen negative people make for the large bulk of population in Western and Central equatorial Africa [20]. Platelet-mediated parasite killing is one of the approaches to protect against malaria; however, the underlying mechanism involving immunity to malaria is incompletely understood. The pathogenic mechanisms of malaria, involving endothelial dysfunction [30], cell-to-cell adhesions mediated by cell-adhesion molecules and aquaporins [30,37,38], inflammatory stimuli released by activated immune cells, and sequestration of iRBC, are complex (Figure S1). Traditional investigations on the pathogenic mechanism of cerebral malaria, such as brain microvascular endothelial cells culture models, membrane permeability assays, transwell systems and animal models, are poorly understood due to restrictions on replicating complicated cell-to-cell signalling, absence of unique brain microvascular features, or interspecies differences [30]. 3D bioprinting approaches remain some challenges, while this technological application will be the *in vitro* studies of cerebral malaria in the future.

In aggregate, our study characterized, for the first time, cell-to-cell platelet–iRBC interactions in participants infected with *P. falciparum*, *P. ovale*, *P. vivax* or *P. malariae*, as well as demonstrated platelet-associated parasite killing and the relationship between platelet and parasite clearance. Given platelet-associated parasite killing, and the risk of longer duration of parasite clearance associated with thrombocytopenia and morphologic alterations of platelets are corroborated, a promising prognostic indicator, platelets, in all four major *Plasmodium* species: *P. falciparum*, *P. ovale*, *P. vivax* or *P. malariae*, infection should be taken into consideration.

## Supplementary Material

Supplemental Material

Supplemental Material

## Data Availability

Clinical, laboratory, treatment and outcome information were gathered from hospital electronic health care records using data collection forms. The blood smears are kept in the department of clinical laboratory. Any further questions should be directed to the corresponding author.
