# Supplementary figures and images for: Clinical characteristics of platelet-mediated killing circulating parasite of major human malaria
Source: Ann Med. 2023 Jun 13;55(1):2221453. doi: 10.1080/07853890.2023.2221453 (PMC10266116; doi:10.1080/07853890.2023.2221453)

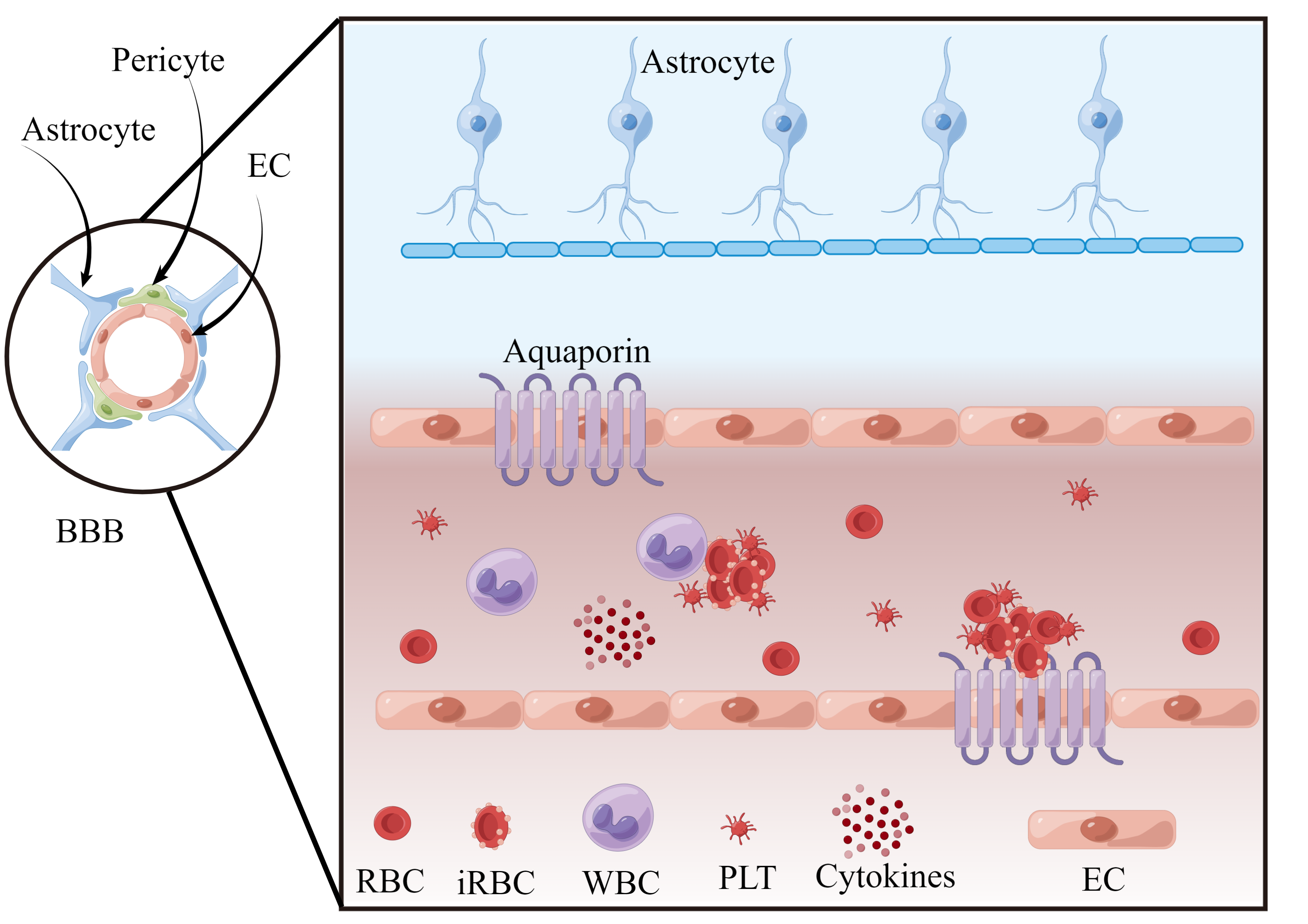

Supplement: Supplemental Material [file IANN_A_2221453_SM3397.tiff]
