# Supplementary material for: Clinical characteristics of platelet-mediated killing circulating parasite of major human malaria
Source: Ann Med. 2023 Jun 13;55(1):2221453. doi: 10.1080/07853890.2023.2221453 (PMC10266116; doi:10.1080/07853890.2023.2221453)
Supplement: Supplemental Material [file IANN_A_2221453_SM1997.docx]

**Figure S1 Mechanism of cerebral malaria** Cerebral malaria pathophysiology develops as a consequence of parasite infection, endothelial cell impairment, blood brain barrier (BBB) disruption, and neuronal injury. These causes an increase in the insulin release of inflammatory cytokines, an increase in the expression of cellular adhesion molecules, the release of hemozoin from ruptured iRBCs, and a decrease in the BBB. Red blood cell, RBC; White blood cell, WBC; Platelet, PLT; Endothelial cells, EC
